# Patients Lacking Sustainable Long-Term Weight Loss after Gastric Bypass Surgery Show Signs of Decreased Inhibitory Control of Prepotent Responses

**DOI:** 10.1371/journal.pone.0119896

**Published:** 2015-03-16

**Authors:** Pleunie S. Hogenkamp, Magnus Sundbom, Victor C. Nilsson, Christian Benedict, Helgi B. Schiöth

**Affiliations:** 1 Department of Neuroscience, Uppsala University, Uppsala, Sweden; 2 Department of Surgical Sciences, Uppsala University, Uppsala, Sweden; University of Florida, UNITED STATES

## Abstract

**Background:**

A considerable number of bariatric patients report poor long-term weight loss after Roux-en-Y gastric bypass (RYGB) surgery. One possibility for an underlying cause is an impairment of cognitive control that impedes this patient group’s dietary efforts.

**Objective:**

To investigate if patients having either poor or good weight loss response, ~12 years after RYGB-surgery, differ in their ability to inhibit prepotent responses when processing food cues during attentional operations—as measure of cognitive control.

**Methods:**

In terms of weight loss following RYGB-surgery, 15 ‘poor responders’ and 15 ‘good responders’, matched for gender, age, education, preoperative body mass index, and years since surgery, were administered two tasks that measure sustained attention and response control: a go/no-go task and a Stroop interference task; both of which are associated with maladaptive eating behaviours.

**Results:**

The poor responders (vs. good responders) needed significantly more time when conducting a go/no-go task (603±134 vs. 519±44 msec, p = 0.03), but the number of errors did not differ between groups. When conducting a Stroop interference task, poor responders read fewer inks than good responders (68±16 vs. 85±10 words, p = 0.002).

**Conclusion:**

Patients lacking sustainable weight loss after RYGB-surgery showed poorer inhibitory control than patients that successfully lost weight. In the authors’ view, these results suggest that cognitive behavioral therapies post-RYGB-surgery may represent a promising behavioral adjuvant to achieve sustainable weight loss in patients undergoing this procedure. Future studies should examine whether these control deficits in poor responders are food-specific or not.

## Introduction

Studies have consistently demonstrated that bariatric surgery represents a powerful means to achieve massive weight loss in severely obese patients [1] [2]. Roux-en-Y gastric bypass (RYGB) surgery significantly reduces food intake and shortens the gastric residence time of ingested food [3] [4]. The markedly reduced functional volume of the stomach, as well as the exclusion of the passage through the duodenum and proximal small bowel, most likely represents the major peripheral mechanisms through which RYGB-surgery induces weight loss in obese humans. However, additional mechanisms have been suggested, such as enhanced thermic effect of food [5], alternations in bile acids or gut microbiota [6] and different effects on the central nervous system (CNS), such as a reduced drive to consume energy-dense food [7] [8]. Studies utilizing either resting or food-related functional magnetic resonance imaging (fMRI) revealed obesity-associated brain activity patterns (e.g. [9] [10] [11] [12]). This suggests that the food intake-lowering effects of RYGB-surgery may partly derive from altered CNS processing of food cues. For instance, obese females treated with RYGB-surgery showed a hypothalamic blood-oxygen level dependent (BOLD) signal to food images that was comparable to that of normal-weight females, although these patients were significantly heavier than the control group [9]. In contrast, obese females who did not undergo RYGB-surgery exhibited a stronger hypothalamic activation to food cues [9]. The hypothalamus is involved in regulating food intake and body weight [13]. Moreover, reduced brain activity following RYGB was observed in dorsolateral prefrontal cortex (dlPFC), an area associated with inhibitory activation [11] and areas of the mesolimbic reward pathway [12]. These CNS systems play an important role for the drive to consume both palatable food and drugs [14].

While our understanding of the mechanisms through which RYGB-surgery induces weight loss has grown in recent years, it is still unclear as to why a considerable number of RYGB-surgery-treated patients do not reach sustainable weight loss in the long-term [15]. It could be that this patient group has difficulties to suppress their drive to eat when exposed to food stimuli, i.e. to inhibit their control. Thus, the present study aimed at investigating of the performance of two tasks that assess inhibitory control or response interference control (ie. Go/no-go task and Stroop interference task) differ between female patients who did not maintain weight after RYGB-surgery (poor responders) and those who successfully kept their weight after the procedure (good responders). Interestingly, a poor performance on these tasks was previously associated with higher body mass in non-patient populations [e.g. [16] [17] [18].

## Methods

### Participants

Fifteen female patients with a good weight-loss response (‘good responders’) and 15 female patients with a poor weight-loss response after RYGB-surgery (‘poor responders’) were enrolled from a population of patients who underwent primary RYGB-surgery at the University Hospital of Uppsala 1993–2003 [19]. During the surgery, the largest part of the stomach was divided and a small proximal gastric pouch of 20–30 ml anastomosed to a 70-cm Roux-limb of proximal jejunum. After reconnecting the 30-cm of the most proximal small bowel, the biliopancreatic limb, into the Roux-limb, the flow of bile and pancreatic juice was restored [2].

Poor responders were defined as patients with an excessive weight and BMI loss of <50% (between 9–15y after surgery) and body mass index (BMI) >30 kg/m^2^ [20]; good responders as patients with an excessive weight and BMI loss of >75% and BMI <30 kg/m^2^. All patients were free from psychoactive drugs and ongoing psychiatric treatment. Responders were pair-wise matched with poor responders for preoperative BMI (±2 BMI units), preoperative weight (±10 kg) and age (±5 years). **Table 1** shows the participants’ characteristics.

**Table 1:** Characteristics (mean ± SD) of female Roux-en-Y gastric bypass patients with a poor weight loss response after surgery (poor responders) and of patients with a good weight loss response (good responders).

	Poor responders (n = 15)		Good responders (n = 15)	
	Mean	SD	Mean	SD
Preoperative weight (kg)	**120**	9.2	**121**	11.9
Preoperative BMI (kg/m^2^)	**43.8**	4.0	**43.9**	3.5
Weight loss (kg) 1 yr after surgery	**33**	10.5	**36**	9.6
BMI loss (kg/m^2^) 1 yr after surgery	**12.0**	3.7	**13.6**	3.5
Years since surgery	**13**	2.6	**12**	2.9
Current weight (kg)	107 *	10.7	82 *	18.4
Current BMI (kg/m^2^)	38.9 *	3.4	29.6 *	5.5^#^
Current age (yrs)	**49**	8.6	**49**	8.9
Weight loss during follow-up (kg)	13 *	12.1	39*	15.7
BMI loss during follow-up (kg/m^2^)	4.9 *	4.6	14.3 *	5.7
Educational level (primary school/ secondary school/university, n/n/n)	**4/5/6**		**4/6/5**	

BMI = body mass index

* Difference between poor responders and good responders (p<0.0001)

^#^ Patients were invited based on BMI 10 yrs after surgery, as collected by Edholm et al [18]. Two of the patients who had a BMI <30 10 years after RYGB-surgery (BMI 27.2 and 29.3 kg/m^2^), but gained weight till the start of the current study, resulting in a BMI >30 when they visited the research centre (BMI 30.8 and 35.7 kg/m^2^, respectively). All but one of the good responders reported a BMI between 28 and 30 kg/m^2^. This one participant had a particular lower BMI.

Participants visited the research centre to conduct the tests as described below. Most visits were scheduled in the afternoon in a fasted state, i.e. at least 1.5 hrs after lunch. For logistic reasons, data of two poor responders and 2 good responders were collected in a sated state.

### Ethics Statement

The present study was approved by the Regional Ethical Review Board in Uppsala, and the procedures followed were in accordance with the Helsinki Declaration. All participants gave written informed consent, and received monetary compensation for their efforts.

### Go/no-go task

A go/no-go association task [16] [18] [21] [22] was used to assess the inhibition of prepotent responses to food items. In this task, words of non-food items (go-trial) or words of food items (no-go-trial) were presented for 500 ms (based on [16]). The task consisted of 10 blocks containing 16 words. Trials (i.e. presence of words) were separated by a fixation cross. Subjects were instructed to respond with a button press to all non-food words, but to withhold this response to food-words, and to respond as quickly and accurately as possible. By having more go-trials (75% occurrence) than no-go trials (25% occurrence), responding rather than inhibiting was made prepotent [15]. We analyzed response times for correct trials and errors. The task was presented on a laptop using Presentation (Version 9, Neurobehavioral Systems, Davis).

### Stroop task

Response interference control was measured using a modified version of a Stroop colour-naming interference task [22] [23] [24]. Participants were presented a series of food-related words printed in different colors. They were instructed to name the color in which each word was printed as quickly as possible, i.e. to inhibit the prepotent reading response. The total number of correct responses within 60 s was determined.

### Questionnaires: TFEQ and BIS-11

The 21-item Three Factor Eating Questionnaire (TFEQ-R21) was used to assess uncontrolled- eating, restraint-eating, and emotional-eating [25], and the 30-item Barratt Impulsivity Scale (BIS-11) to measure total impulsiveness, as well as motor, non-planning and attentional impulsiveness [26]. Higher scores indicate more uncontrolled, restraint and emotional eating; or higher impulsiveness levels.

Educational level was assessed using a categorized questionnaire (‘primary school’ (9y in school), ‘secondary school’ (9–12y), and ‘university or higher’ (>12y)).

### Data analysis

Data are presented as means (±SD) unless otherwise indicated. Differences in performance on the tasks between poor responders and good responders were tested using independent t-tests. Associations between measures and weight loss were explored using Pearson's correlation, and a linear regression model was used to adjust for educational status, as a proxy of intellectual capacity. Data were analyzed using SPSS software (SPSS Inc, Chicago, Ill).

## Results

**Fig 1:**
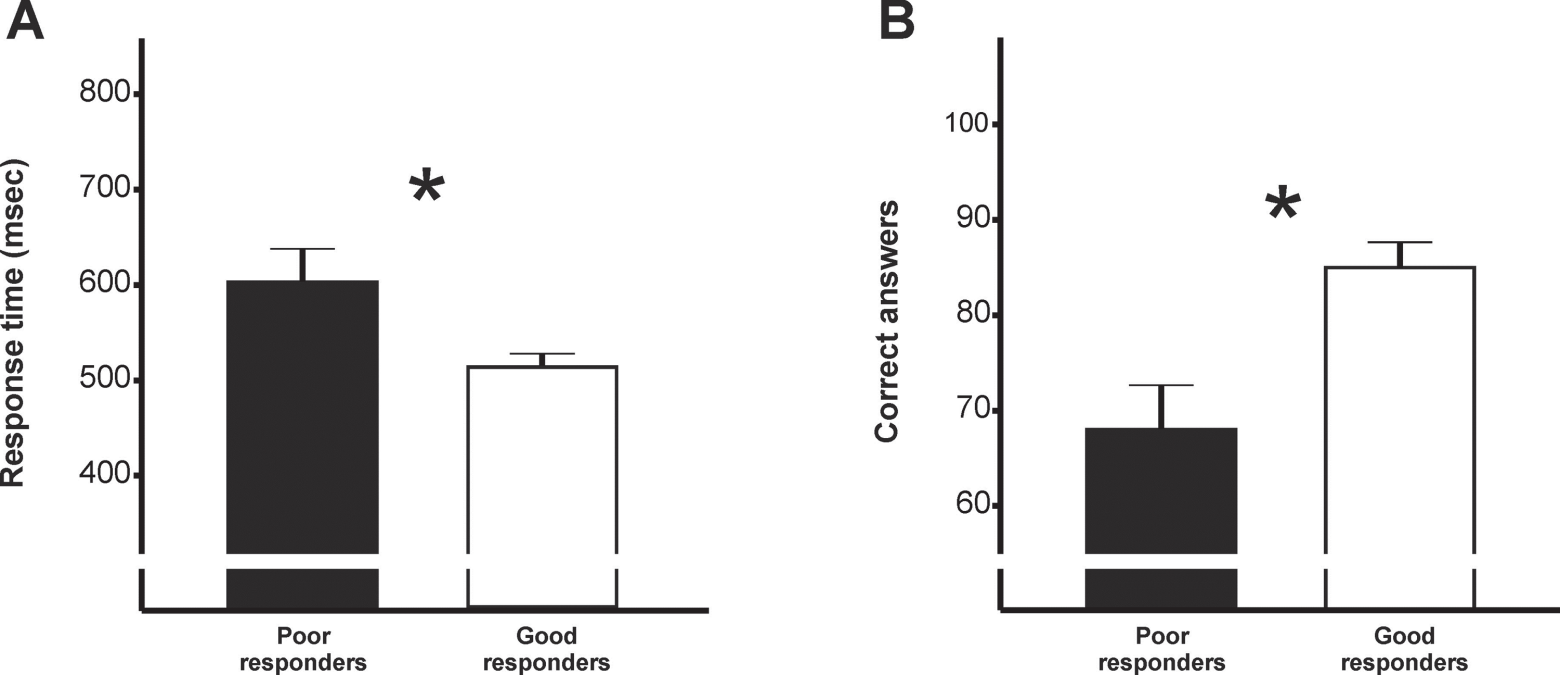
Performance on go/no-go task and Stroop interference task by patients who showed a poor weight loss response to gastric bypass (RYGB) surgery ~ 12 years after surgery (poor responders, n = 15, black bars) and patients who responded well to RYGB-surgery ~ 12 years after surgery (good responders, n = 15, white bars). **(A)** Average response time to ‘go’ stimuli (i.e. non-food-related words as opposed to food words as ‘no-go’ stimuli). **(B)** Performance on the food-related Stroop interference task. All groups were matched for pre-operative weight/BMI, educational status and age (as shown in **Table 1**). * P<0.05 for significant differences between poor and good responders.

In the go/no-go task the average response time to ‘go’ stimuli differed across groups: poor responders were slower than good responders; t = 2.37, p = 0.03; **Fig. 1A**). The number of commission errors (responding when presented ‘no-go’ stimuli) and omission errors (withholding responses when presented ‘go’ stimuli) did not differ across groups: poor vs. good responders made 7.5±5.5 vs. 8.1±3.2 commission errors and 0.9±1.6 vs. 1.8±0.9 ommission errors. Stroop task performance also differed across groups: poor responders performed worse than good responders (t = -3.39, p = 0.002; **Fig. 1B**).

Scores on TFEQ-subscales, BIS-11 impulsivity, and BIS-11 subscales were not different between good and poor responders. However, scores for uncontrolled eating (TFEQ) were associated with inferior weight loss (kg) ~12 years after surgery (r = -0.41, p = 0.025). A similar trend was seen for attentional impulsiveness (BIS-11) and weight loss (r = -0.35, p = 0.067). Inclusion of educational level as a covariate in our analyses did not change the observed differences or associations.

## Discussion

Our study demonstrates that female patients lacking sustainable weight loss approximately 12 years after RYGB-surgery (‘poor responders’) performed worse on the go/no go task and Stroop task, when compared to patients with a good sustainable weight after RYGB-surgery (‘good responders’). These results suggest that food cues possess a distracting value for poor responders after the surgery. Moreover, both BIS attentional scores and TFEQ uncontrolled eating scores were negatively associated with post-surgery weight loss. Taken together, these results suggest that cognitive traits related to eating behavior may impact surgery-induced weight loss response. Therefore, future studies should examine whether these control deficits in poor responders are food-specific, and to which extent cognitive behavioral therapeutic strategies targeting cognitive control over food cues may help achieve a sustainable normal weight in patients undergoing RYGB-surgery.

Performances on the tasks have previously been associated with various maladaptive eating behaviors [22]. Compared to normal-weight individuals, obese people perform worse when the task includes food stimuli [22] [27], and the magnitude of inhibitory control in response to food cues is linked to future weight gain [16]. This is also supported by an overview of fMRI studies comparing obese vs. lean individuals, which suggests that diminished functioning of inhibitory control regions and abnormal responses of food reward regions are associated with elevated weight [28]. In line with this, fMRI studies in bariatric patients demonstrate that increased neural activity in frontal regions associated with cognitive control is associated with weight loss 3 and 6 months after RYGB-surgery [10] and also with reduced activation in brain areas that are important for motivation and food reward after both RYGB-surgery [12] and gastric banding surgery [29]. At this point it is important to mention that our study does not examine if poor responders would also perform poorly on tasks not involving food stimuli. In other words, there is the possibility the observed group differences were prompted by a general lower inhibitory capacity among poor responders.

Another potential mechanism could relate to physiological consequences of RYGB-surgery, such as improved insulin sensitivity and increased circulating levels of glucagon-like peptide-1 (GLP-1) and PYY in response to food [30] [31]. These hormones influence CNS functions, including food-reward processing, cognitive control over eating, and higher cognitive functions (e.g. [31] [32] [33]). Patients with a poor weight loss response to RYBG-surgery show sub-optimal GLP-1 responses, and they are more insulin resistant compared to those with good weight loss responses [34]. Although speculative, possibly the impact of ingested food on the release of the above-mentioned hormones is less pronounced in poor responders. As a result, RYGB-surgery may strengthen cognitive control over eating to a lesser extent under postprandial conditions in poor responders [31]. However, long-term prospective studies which include hormonal aspects of reward functioning will be needed to validate this hypothesis.

### Strengths, limitations and perspectives

A strength of our study is that patient groups (poor vs. good responders) were matched for gender, age, education, and pre-operative body weight and BMI. A second strength is the long follow-up period (~12 years). The main limitation of our study is that cognitive functions were not tested prior to surgery, i.e. we cannot estimate if the observed group differences in inhibitory control were surgery-dependent or not. However, supporting the view that surgery may alter cognitive functions in favor of weight loss, a recent study demonstrated that cognitive functions assessed shortly after surgery were associated with weight loss up to 36 months, e.g.[35]. Moreover, entering educational level in our analysis—as a proxy of both intellectual function and socioeconomic status [36]—did not change the result pattern, but we could not control for other factors that may affect both body weight and psychological function, such as physical activity. This indicates that pre-surgery differences in acquired intellectual properties between poor and good responders are unlikely to play a major role for the observed effects. However, we did not control for other factors that may affect post-surgery body weight and psychological function, e.g. physical activity. Finally, the relatively small sample size of our study must be borne in mind when extrapolating our results to patients undergoing bariatric surgery.

## Conclusion

Patients with a poor long-term weight loss response after RYGB-surgery performed worse on the go/no-go task and the Stroop task than patients who did successfully maintain weight loss. The results of the present study provide a rationale for hypothesizing that post-RYGB surgery therapies targeting cognitive control may represent a promising behavioral adjuvant to achieve sustainable weight loss in patients undergoing this procedure. Future studies should examine if these control deficits in poor responders are food-specific or not.

## Supporting Information

S1 Table Data—patients who showed a poor or a good weight loss response to gastric bypass (RYGB) surgery ~ 12 years after surgery. (PDF)
