# Supplementary material for: Patients Lacking Sustainable Long-Term Weight Loss after Gastric Bypass Surgery Show Signs of Decreased Inhibitory Control of Prepotent Responses
Source: PLoS One. 2015 Mar 16;10(3):e0119896. doi: 10.1371/journal.pone.0119896 (PMC4361610; doi:10.1371/journal.pone.0119896)
Supplement: S1 Table — (PDF) [file pone.0119896.s001.pdf]

ID	Good Responder=1	Pre-surg. BMI	Pre-surg. weight kg	Current weight kg	current BMI	Education	Followup yrs	Current age	weightloss kg 1yr
201.0	.0	35.6	109	104.4	34.1	3.0	15.0	42	40
202.0	.0	37.9	107	97.2	34.0	2.0	15.0	55	31
203.0	.0	39.7	116	122.0	42.7	1.0	14.0	51	21
204.0	.0	41.7	108	101.5	39.6	2.0	15.0	46	23
205.0	.0	42.5	120	114.9	41.7	3.0	15.0	45	45
206.0	.0	43.1	113	104.0	38.2	1.0	12.0	65	34
207.0	.0	46.4	131	128.0	45.4	1.0	10.0	61	
208.0	.0	45.5	124	104.4	38.3	2.0	15.0	58	46
209.0	.0	46.3	126	92.4	33.9	3.0	15.0	40	38
210.0	.0	46.7	135	119.4	41.3	2.0	12.0	40	50
211.0	.0	47.8	130	110.6	40.6	3.0	15.0	46	30
212.0	.0	49.8	129	89.5	35.4	3.0	11.0	43	36
213.0	.0	41.9	110	107.2	40.8	1.0	15.0	42	
214.0	.0	44.1	120	103.6	38.1	2.0	9.0	62	16
215.0	.0	48.3	122	100.9	39.9	3.0	8.0	46	23
1101.0	1.0	38.0	106	46.6	16.7	3.0	13.0	41	37
1102.0	1.0	37.8	103	83.0	30.9	1.0	16.0	62	33
1103.0	1.0	43.0	110	74.9	29.3	2.0	10.0	48	27
1104.0	1.0	41.4	114	85.9	31.2	1.0	10.0	44	22
1105.0	1.0	42.9	130	99.4	32.5	3.0	10.0	44	39
1106.0	1.0	42.7	119	73.9	26.5	1.0	14.0	63	31
1107.0	1.0	45.1	107	68.9	29.8	1.0	12.0	60	40
1108.0	1.0	45.5	130	86.5	29.9	2.0	14.0	51	
1109.0	1.0	46.5	125	71.0	26.1	2.0	14.0	39	49
1110.0	1.0	46.1	130	106.8	37.4	3.0	10.0	35	
1111.0	1.0	47.8	130	81.4	29.9	3.0	15.0	51	35
1112.0	1.0	49.8	139	65.7	23.6	2.0	13.0	45	58
1113.0	1.0	42.0	139	118.3	35.7	3.0	11.0	52	
1114.0	1.0	42.5	113	69.7	26.2	2.0	6.0	62	33
1115.0	1.0	48.0	123	101.7	38.3	2.0	7.0	44	33

ID	BMIloss 1yr	weightloss_kg followup	BMIloss followup	TFEQ Uncontr. Eating 1	TFEQ Cogn. restraint 1	TFEQ Emot. eating 1	TFEQ uncontr. Eating 2
201.0	13.1	4.6	1.5	2.22	2.33	2.00	.41
202.0	11.3	9.8	3.9	2.22	2.33	2.33	.41
203.0	6.4	-6.0	-3.0	2.33	1.67	3.50	.44
204.0	8.5	6.5	2.0	1.00	1.50	1.00	.00
205.0	15.3	5.1	.8	2.33	2.00	3.67	.44
206.0	14.0	9.0	4.9	2.22	2.17	2.83	.41
207.0		3.0	1.0	2.89	2.83	2.33	.63
208.0	16.9	19.6	7.2	2.22	2.33	2.83	.41
209.0	14.0	33.6	12.3	1.33	2.67	1.17	.11
210.0	17.3	15.6	5.4	1.44	2.17	2.67	.15
211.0	11.0	19.4	7.1	2.33	2.33	2.00	.44
212.0	13.0	39.5	14.4	2.22	2.50	2.33	.41
213.0		2.8	1.1	1.22	2.50	1.00	.07
214.0	5.9	16.4	6.0	2.11	3.33	2.83	.37
215.0	9.1	21.1	8.4	1.78	3.00	1.50	.26
1101.0	13.3	59.4	21.3	1.00	1.00	1.00	.00
1102.0	11.8	20.0	7.0	1.56	1.67	1.50	.19
1103.0	10.5	35.1	13.7	2.33	1.67	2.67	.44
1104.0	8.0	28.1	10.2	2.22	2.17	1.67	.41
1105.0	13.2	30.6	10.5	2.89	2.33	3.83	.63
1106.0	11.1	45.1	16.2	1.22	2.00	1.17	.07
1107.0	16.1	38.1	15.3	1.89	1.83	2.67	.30
1108.0		43.5	15.6	2.38	3.00	3.50	.37
1109.0	18.6	54.0	20.4	1.11	1.67	1.00	.04
1110.0		23.2	8.7	2.11	3.17	2.33	.37
1111.0	12.9	48.6	17.9	1.44	1.83	1.67	.15
1112.0	20.8	73.3	26.3	1.44	2.17	2.17	.15
1113.0		20.7	6.2	1.67	3.17	2.00	.22
1114.0	12.4	43.3	16.3	1.11	1.83	1.33	.04
1115.0	14.1	21.3	9.7	3.22	1.83	2.83	.74

ID	TFEQ Cogn. restraint 2	TFEQ Emot. eating 2	Stroop # words	BIS11 Attention	BIS11 Motor	BIS11 Nonplann.	BIS11 total	average RT Go NoGo msec	Comm. errors	Omm. errors
201.0	.44	.33	46.0	13	19	23	55	520.6	8.0	.0
202.0	.44	.44	56.0	15	18	22	55	817.5	1.0	.0
203.0	.22	.83	79.0	18	20	27	65	526.7	1.0	1.0
204.0	.17	.00	84.0	16	20	16	52	513.6	14.0	6.0
205.0	.33	.89	58.0	16	16	23	55	460.8	13.0	2.0
206.0	.39	.61	54.0	12	20	18	50	839.2	8.0	1.0
207.0	.61	.44	50.0	23	27	25	75	634.3	15.0	1.0
208.0	.44	.61	72.0	14	19	24	57	633.3	.0	.0
209.0	.56	.06	60.0	11	19	25	55	527.4	10.0	1.0
210.0	.39	.56	83.0	13	19	26	58	498.1	14.0	.0
211.0	.44	.33	82.0	18	23	28	69	443.6	12.0	.0
212.0	.50	.44	90.0	14	21	17	52	710.2	4.0	.0
213.0	.50	.00	55.0	20	17	18	55	556.6	10.0	.0
214.0	.78	.61	57.0	15	20	21	56	820.7	1.0	1.0
215.0	.67	.17	98.0					546.5	2.0	.0
1101.0	.00	.00	85.0	12	20	16	48	539.8	6.0	.0
1102.0	.22	.17	78.0	13	20	17	50	584.9	7.0	.0
1103.0	.22	.56	89.0	17	25	31	73	496.4	5.0	.0
1104.0	.39	.22	100.0	12	19	20	51	498.2	6.0	5.0
1105.0	.44	.94	80.0	12	16	19	47	515.3	4.0	.0
1106.0	.33	.06	77.0	14	17	22	53	599.3	3.0	.0
1107.0	.28	.56	75.0	19	18	34	71	497.5	9.0	1.0
1108.0	.67	.83	100.0	16	20	26	62	487.2	13.0	1.0
1109.0	.22	.00	72.0	10	23	23	56	534.6	11.0	.0
1110.0	.72	.44	100.0	14	20	26	60	531.4	11.0	.0
1111.0	.28	.22	71.0	17	18	27	62	735.3	1.0	.0
1112.0	.39	.39	87.0	14	16	29	59	523.8	12.0	8.0
1113.0	.72	.33	82.0	13	20	20	53	451.8	11.0	7.0
1114.0	.28	.11	86.0	16	23	24	63	501.3	7.0	3.0
1115.0	.28	.61	95.0	18	31	34	83	439.4	9.0	.0
